# Donor site morbidity in oral mucosa graft urethroplasty: implications of tobacco consumption

**DOI:** 10.1186/1471-2490-9-15

**Published:** 2009-09-21

**Authors:** Rahul Janak Sinha, Vishwajeet Singh, SN Sankhwar, Divakar Dalela

**Affiliations:** 1 Department of Urology, Muljibhai Patel Urological Hospital, Nadiad, India; 2 Department of Urology, CSMMU (formerly KGMU), Lucknow, (U.P.), India

## Abstract

**Background:**

The purpose of this prospective study was to evaluate the donor site morbidity in patients who have undergone oral mucosa graft urethroplasty for stricture of the urethra. The impact of smoking and oral consumption of tobacco and/or *paan masala *on the donor site was also assessed. This study is probably the first of its kind where the affect of smoking, *paan masala *and tobacco chewing on the donor site morbidity has been documented.

**Methods:**

Forty-eight patients suffering from stricture of the urethra underwent oral mucosa graft urethroplasty between July 2005 and December 2007. The patients were divided into two groups (users or non-users) based on tobacco consumption and oral hygiene. The donor site was evaluated at frequent intervals for pain, swelling, numbness, bleeding, salivation and tightness of mouth.

**Results:**

Donor site morbidity was more in users with poor oral hygiene. Pain scores were higher amongst the users and the morbidity persisted longer in the users compared to non-users with good oral hygiene.

**Conclusion:**

Patients who consume tobacco and have poor oral hygiene should be warned regarding poorer outcomes after oral mucosa graft urethroplasty.

## Background

Consumption of *paan *(betel leaves) or *paan masala *(dried mixture of betel leaves with areca nut and slaked lime which is consumed with or without tobacco along with other condiments) is one of the unique social customs prevalent in South - East Asia. [1,2] Unfortunately this habit leads to deterioration in the quality of oral mucosa. Many of the patients, who visit this institute for treatment, consume tobacco in various forms and/or smoke. The quality of oral mucosa is compromised by the consumption of tobacco and smoking. [1-4] This may lead to increased donor site morbidity after oral mucosa graft harvest.

The purpose of this study was to evaluate the donor site morbidity in patients who underwent oral mucosa graft urethroplasty (OMGU) for stricture of the urethra. This prospective study is probably the first of its kind where the impact of smoking, tobacco chewing and *paan masala *consumption on donor site morbidity is being documented.

## Methods

A prospective study was performed which included 48 patients who underwent OMGU between July 2005 and December 2007. The last follow-up of these patients was till June 2008. Written informed consent was obtained from all the patients included in this study. Ethical clearance for this study was obtained from the institutional ethics committee and was in accordance with the Declaration of Helsinki. Patients were divided into two groups - users - those who consumed tobacco in any form and had a poor oral hygiene and non-users - those who did not consumed tobacco in any form and had a good oral hygiene. General data of all the patients e.g. name, age, sex, address and phone number were recorded for the purpose of identification and correspondence. Routine laboratory and specific radiological evaluation was performed prior to surgery.

Oral hygiene was inspected by the authors and those with poor oral hygiene were sent to the dental department for further opinion. Graft from these patients was only harvested if the dental department cleared these patients for OMGU. Distribution of the patients according to their type of addiction is mentioned in Table 1. Intra-operative variables related to the donor site are mentioned in Table 2. Duration of exposure to tobacco was assessed in the users and documented in Table 3. Donor site morbidity at 48 hours, at 1 week, at 1 month, at 6 months and at 1 year respectively was the outcome considered for this study. Parameters used were post-operative bleeding, pain, swelling, tightness, numbness or more than one of the above mentioned morbidities (Table 4). Visual analogue scale (VAS) was used to compare the pain score between the two groups.

**Table 1:** Distribution of users according to the type of tobacco consumption

**Type of addiction**	**Number of patients**	**Percentage (%)**
*Paan masala*	3	10.7

Tobacco chewing	3	10.7

Smoking	3	10.7

*Paan Masala *with tobacco chewing	6	21.4

*Paan Masala *with smoking	4	14.3

Tobacco chewing and smoking	3	10.7

*Paan Masala *with tobacco chewing and smoking	6	21.4

Total no. of addicts	28	100.0

**Table 2:** Graft harvest details

**Indicators**	**Variable**	**Users**	**Non-users**
Type of graft	Patch	28	20

Donor site	Only One Cheek	14	10
	
	Both Cheeks only	11	7
	
	Both Cheeks + Tongue	2	1
	
	Both Cheeks + Lower Lip	1	2
	
	Total	28	20

Donor site treatment following graft Harvest	Unstitched	24	16
	
	Stitched	4	4
	
	Total	28	20

**Table 3:** Distribution of users according to the duration of exposure to tobacco

**Type of addiction**	**Number of patients**	**Exposure for 5 years or less**	**Exposure for more than 5 but less than 10 years**	**Exposure for more than 10 years**
*Paan masala*	3	1	1	1

Tobacco chewing	3	1	1	1

Smoking	3	1	1	1

*Paan Masala *with tobacco chewing	6	3	2	1

*Paan Masala *with smoking	4	2	1	1

Tobacco chewing and smoking	3	1	1	1

*Paan Masala *with tobacco chewing and smoking	6	4	1	1

No. of addicts	28 (Total)	13	8	7

**Table 4:** Donor site morbidity at different time intervals during follow up

**Symptoms**										
**Duration of follow up**	**At 48 hours**		**At 1 week**		**At 1 month**		**At 6 months**		**At 1 year**	
	
	**Users**	**Non-users**	**Users**	**Non-users**	**Users**	**Non-users**	**Users**	**Non-users**	**Users**	**Non-users**

Postoperative bleeding from the donor site	8	2	2	0	0	0	0	0	0	0

Pain at the graft harvest site	22	12	16	6	9	2	4	1	2	0

Swelling of the graft harvest site	16	4	10	1	4	0	2	0	0	0

Tightness of mouth (difficulty in opening the mouth)	9	2	5	2	4	1	3	0	2	0

Numbness of the graft harvest site	7	4	7	2	6	1	5	1	5	0

More than one morbidity co-existing in the same patient	17	6	12	3	7	0	5	0	3	0

Patients having no morbidity	4	6	8	14	14	16	14	11	9	10

Total	28	20	28	20	28	20	19	13	14	10

### Procedure of graft harvest

Patients are instructed to use a mouthwash containing chlorhexidine in the pre-operative period. All the patients receive intra-operative antibiotics (Ceftriaxone and Sulbactam combination, Amikacin and Metronidazole) intravenously before the oral mucosa is excised. Initially 12 patients were operated under general anesthesia (nasal or oral intubation) but at present the graft is harvested under local analgesia according to our technique [5] and all the patients are operated under regional anesthesia (epidural or spinal). [6,7] The patients are counseled regarding the graft harvest in the pre-operative period and any queries raised by them regarding the procedure is answered. This helps as the patients cooperate better during the graft harvest. Two surgical teams work simultaneously, each having its own set of instruments.

In the majority, the graft is excised from the cheek; the donor site was stitched in the initial few cases (Table 2) but now it is left unstitched as a routine practice. [8-10] Donor site is packed with a gauze piece soaked in adrenaline and lignocaine. The oral pack is removed in the evening and the patient is asked to rinse his mouth with cold water and dilute mouthwash. The cavity is inspected for any bleeding and the patient is asked to start cold oral liquids in the evening. In a day or two the patient is advised to shift to semi-solid, non-spicy diet and can consume normal diet as soon as he can tolerate it.

Phone calls and letters were used to enquire about the general well being of the patients. Even though only 42.1% of the patients responded to phone calls while 54% responded to the letter; still the majority came for follow-up irrespective of the phone calls or letters. A proforma was prepared at the time of admission, which documented all the raw data. Follow-up data was collected by an interviewer. The interviewer understood the significance and meaning of the questions and asked the questions in *Hindi *language which is the spoken language in the northern India.

### Statistical Analysis

The data was entered in the MS-Excel computer program and all the analyses were carried out using SPSS (Ver.15.0) statistical program. The mean and standard deviations were calculated for continuous variables such as age, different lengths variables and proportions (percentages) were calculated for discrete variables.

The Chi-square test was used to compare dichotomous/categorical variables.

The paired t-test was used to detect significance from baseline value to follow-up time in case of continuous variables and unpaired t-test was used to detect the difference between two continuous variables.

Proper checks were made to check the normality of the data and all the continuous parameters were found to be normally distributed. Hence, the parametric test is being used for these parameters. The p-value < 0.05 was considered as significant.

## Results

The post-operative follow-up of these 48 patients at one month was 100% since all the patients came back for catheter removal and all had completed their follow-up of 1 month. Following that 3 patients (2 users and 1 non-user) were lost to follow-up at 6 monthly follow up and at 1 year follow-up 6 patients (4 users and 2 non-users) could not be accounted for. So further follow-up was assessed in these 42 patients; donor site morbidity at 6 months was assessed in 32 patients and 1 year follow-up in 24 of these patients who matured to that stage of follow-up at that given time.

Symptoms related to donor site were assessed within 48 hours of surgery, then at 1 week, 1 month, 6 months and 1 year after the surgery (Table 4).

Eleven patients had co-morbid conditions prior to the surgery but that did not impact the donor site morbidity during intra-operative or post-operative period. Intra-operative complications related to the donor site was rare - one user patient with unstable dentures had tooth dislodgement during oral intubation.

The mean age of all the patients (n = 48) was 36.60 ± 16.93 years (range 12 - 72 years) while that of the users (n = 28) was 40.24 ± 14.68 (range 22 - 72 years) and non - users (n = 20) was 32 ± 18.24 (range 12 - 68 years). Mean follow-up of these patients was 18.2 months (range 6 - 36 months). Duration of disease in all the patients (n = 48) was 5.77 ± 4.96 years (range 4 months - 15 years) and was similar between users and non-users.

The stricture length (n = 48) was 9.88 ± 5.21 cm (range 2.00 - 17.80 cm). The graft length (n = 48) was 10.42 ± 5.12 cm (range 2.50 - 18.00 cm) and the graft width (n = 48) was 2.62 ± 0.18 cm (range 2.30 - 3.10 cm). The measurements were similar between the users and the non-users.

Donor site morbidity was observed in most of the patients in both the groups at 48 hours; primary symptom being pain followed by swelling, numbness and difficulty in opening the mouth. Post-operative bleeding was more in users as compared to non-users at 48 hours and persisted in 2 of the users even at 1 week while the non-users recovered quickly from this morbidity. Pain at the harvest site was more in users at 48 hours and persisted for a longer duration in the user group signifying greater morbidity. Swelling occurred in the users but subsided after 1 month and only 2 users had swelling at 6 month follow-up. Difficulty in mouth opening and numbness was also more common amongst users and those with poor hygiene and persisted for a very long time. Users were more prone to multiple oral morbidities. As the time interval increased most of the symptoms subsided. Non-users had lower pain scores and faster recovery compared to the users (Table 4).

Differences in oral morbidity between users and non-users were not statistically significant.

## Discussion

Patients in our study had increased donor site morbidity and poor oral recovery if the oral hygiene was compromised to begin with. Even though a few western studies have documented both short and long term donor site morbidity but they have not focused upon oral hygiene or tobacco consumption in any form.

Wood *et al. *[11] assessed the medium and long-term complications via a patient postal questionnaire. In their study, 83% patients experienced postoperative pain at the site of graft harvest. Perioral numbness was noted in their study in 68% of patients, which persisted in 26% at or beyond 6 months of follow-up. Surprisingly, pain was unrelated to size of graft harvest in that study. In our study, patients with a longer or bilateral cheek graft harvest had higher pain scores within the user as well as the non-user group; users had higher pain scores overall and for longer period as mentioned previously.

In another study, Dublin *et al. *[12] found that in the post-operative period the major symptoms were pain, numbness and tightness of the mouth. In their patients, the donor site was sutured which probably led to more pain. Our patients had pain but it subsided quickly because the donor site was left unstitched in 83.3% of the patients.

Jang *et al. *[13] compared post-operative intraoral morbidity after graft harvest from the lower lip and inner cheek. At a longer follow-up, patients whose grafts were harvested from the lower lip had more persistent discomfort, salivary flow changes, and neurosensory deficits than those with cheek harvest. We harvested graft from the lower lip in 3 patients only (1 user and 2 non-users) resulting in lower level of morbidity in non-users. In a similar study Kamp *et al. *[14] evaluated 24 patients and found that graft harvesting from the lower lip led to significantly prolonged discomfort for the patients. These studies reiterate our view that cheek is the best site for oral mucosa harvest since problems like salivary flow changes or cosmetic deformity are not encountered in graft harvest from the cheek as compared to lower lip [15].

Nelson *et al. *[15] also support the view that cheek is the better option for graft harvest and stated that most common complaint in their patients was cosmetic and none of the patients whose oral graft harvest site was limited to the cheek mucosa (as opposed to the lip) had cosmetic complaints. According to the authors, there was no difference between patients whose donor site was closed primarily or allowed to re-epithelize secondarily contraindicates our view that donor site should not be stitched since it causes less pain when left unstitched.

Fabbroni *et al. *[16] assessed the morbidity at the donor site and recorded any problems related to injury to the lingual and mental nerves, symptoms of obstruction of the parotid duct, and trismus but observed only four early complaints of mild trismus and one late complaint. This study again confirms our observations that post-operative donor site morbidity is limited to a small percentage of patients. It was observed in 20% of non-users and in 50% of users at 1 month; 15.4% of non-users and 26% of users at 6 months and 35.7% of users and 0.0% of non-users at 1 year. With the passage of time, most of the patients had milder symptoms. Percentage of users with donor site morbidity was more at 1 year as compared to that at 6 months perhaps due to the fact that the number of users who were recovering better did not come for follow up compared to those who still had co-morbidities.

Markiewicz *et al. *[17] reviewed the literature regarding complications associated with the donor site and found that the most frequent complications at mucosal harvest sites were scarring and contracture which might have been due to the fact that earlier all donor sites were stitched. We did not encounter the above mentioned problems since we left the donor site unstitched in 83.3% of patients as stated earlier.

Dubey *et al. *[18,19] in two separate studies mentioned donor site morbidity in Indian patients. According to the authors, oral complications in the buccal mucosa urethroplasty group were few and of short term duration. Unlike our study, the description of donor site morbidity was brief and did not take the oral hygiene into account.

In a recent study, Castagnetti *et al. *[20] reported short term and long term retrospective assessment of donor site morbidity in a heterogeneous group of patients who underwent oral mucosa graft harvest. At long term assessment 28% of patients had perioral sensory deficit. This deficit was seldom perceived by the patients and was only reported if the oral surgeon examined them. From this study we can infer that long term morbidity after graft harvest is minimal. In our study, numbness at graft harvest site at 6 months amongst users and non-users was 26.3% and 7.7% respectively and that at 1 year was 35.7% and 0.0% respectively. The reason why percentage of numbness increased with increasing time interval is perhaps due to the fact that the patient can appreciate numbness better once pain and swelling subside. The other reason as mentioned above is due to more of such patients coming for follow-up compared to those who were perhaps getting better.

The weakness in our study is that it does not have a long term data and the patient population is small due to which the results of this study failed to reach any statistical significance. Since it is rare to find a person who chews tobacco or *paan masala *and smokes and still has a good oral hygiene; we have not divided the patients further into two more groups *e.g*. non-users with poor hygiene and users with good hygiene due to small number of patients in our study. Nonetheless, we accept this as a limitation of our study. Since oral hygiene of the patient was initially inspected by us and we then decided to refer the patients to the dental department; this step might have introduced a bias in the patient selection and is another limitation of this study. Another limitation of this study could be due to the use of chlorhexidine mouthwash in the pre-operative period which could have reduced the degree of local inflammation, improved the condition of oral mucosa and thus influenced the outcome of this study by reducing the morbidity. We also believe that it is highly unlikely since the mouthwash was used for a short time in the pre-operative period and majority of the patients were consuming tobacco for a very long period of time.

The strength of this study is that it is a prospective study and adds a new dimension to the donor site morbidity in terms of oral hygiene and tobacco consumption. The duration of exposure to tobacco has also been accounted for in our study and the patients have been divided in two groups based on exposure to tobacco.

## Conclusion

In OMGU, donor site morbidity needs to be documented. Patients having poor oral hygiene and those consuming tobacco should be cautioned regarding increased donor site morbidity. A future study might give some indication whether patients having poor oral hygiene or on the borderline can be prepared better with chlorhexidine mouthwash or any other medication and made more acceptable for OMGU. This might expand the indication of OMGU and provide benefit to more patients. More studies (perhaps of longer duration) need to be published which focus on the donor site morbidity in context of tobacco chewing and smoking to reach a valid conclusion.

## Abbreviations

OMGU: Oral mucosa graft urethroplasty; VAS: Visual analogue scale

## Competing interests

The authors declare that they have no competing interests.

## Authors' contributions

RJS treated the patients included in this study, conceived of the study, carried out the research for this study, analyzed the data and prepared the manuscript. VS treated the patients included in this study, corrected the manuscript, added references wherever necessary and revised it critically. SNS treated the patients included in this study, participated in the concept and design of the study, helped with the statistical analysis and gave final approval of the version to be published. DD treated the patients included in this study and participated in its design and coordination. All the authors read and approved the final manuscript.

## Pre-publication history

The pre-publication history for this paper can be accessed here:


